# Overexpression of Different Types of Microbial Rhodopsins with a Highly Expressible Bacteriorhodopsin from *Haloarcula marismortui* as a Single Protein in *E. coli*

**DOI:** 10.1038/s41598-018-32399-x

**Published:** 2018-09-19

**Authors:** Cheng-Hong Tu, Hsiu-Ping Yi, Shiang-Yuan Hsieh, Hong-Syuan Lin, Chii-Shen Yang

**Affiliations:** Department of Biochemical Science and Technology, National Taiwan University, Taipei, 10616 Taiwan

## Abstract

Microbial rhodopsins (M-Rho) are found in Archaea, Bacteria and some species of Eukarya and serve as light-driven ion pumps or mediate phototaxis responses in various biological systems. We previously reported an expression system using a highly expressible mutant, D94N-HmBRI (HEBR) from *Haloarcula marismortui*, as a leading tag to assist in the expression of membrane proteins that were otherwise difficult to express in *E. coli*. In this study, we show a universal strategy for the expression of two M-Rho proteins, either the same or different types, as one fusion protein with the HEBR system. One extra transmembrane domain was engineered to the C-terminal of HEBR to express another target M-Rho. The average expression yield in this new system reached a minimum of 2 mg/L culture, and the maximum absorbance of the target M-Rho remained unaltered in the fusion forms. The fusion protein showed a combined absorbance spectrum of a lone HEBR and target M-Rho. The function of the target M-Rho was not affected after examination with functional tests, including the photocycle and proton pumping activity of fusion proteins. In addition, an otherwise unstable sensory rhodopsin, HmSRM, showed the same or even improved stability under various temperatures, salt concentrations, and a wide range of pH conditions. This HEBR platform provides the possibility to construct multi-functional, stoichiometric and color-tuning fusion proteins using M-Rho from haloarchaea.

## Introduction

Microbial rhodopsins (M-Rho) belong to a group of proteins that feature seven transmembrane regions with retinal bound as their chromophore to sense light. They exert many types of light-triggered functionalities by responding to different specific wavelengths of light, including but not limited to ion translocation^1^ or phototaxis responses^2^.

The ion-translocation type includes bacteriorhodopsin (BR) and halorhodopsin (HR). After HsBR from *Halobacterium salinarum* was identified in 1971^3^, it was soon shown to be a light-driven outward proton pump^4–6^. The later discovery of other microbial rhodopsins, such as the light-driven inward chloride pump halorhodopsin (HR)^7–9^, enriched the functional variation in light-driven ion translocation M-Rho types. The second type includes photosensory rhodopsins (SR)^10–13^, which were found to mediate positive or negative phototaxis responses in haloarchaea. Additional types of M-Rho with different functionalities were later discovered, including fungal rhodopsin (Nop1)^14^, proteorhodopsins (PR)^15^, channelrhodopsins^16^ and a few new types of rhodopsins from marine organisms^2,17^. These findings suggest the global economic importance of M-Rho.

In addition to unveiling the new functions and biophysical properties of those M-Rho proteins, efforts were also exerted to explore their various potential applications. The unique properties of BR, their photochromism and high thermal stability, have been the primary features in physicochemical studies. These include adopting bacteriorhodopsin as a photocurrent generator^18–20^, as well as for various biotechnological apparatuses^21^. The versatility of other M-Rho proteins was also shown in recent developments. For instance, both halorhodopsin (HR), a light-driven inward chloride pump, and light-gated inward cation channel ChR2 were shown to deactivate and activate, respectively, nerve fiber under different wavelengths of light^22^.

To create even more versatile applications, expressing different types of M-Rho as one fusion protein is indeed a logical approach, since it will enable the design of new, multi-capability proteins, which can be fixed at a desired stoichiometric ratio of component moieties. In fact, a study^23^ showed stoichiometric and co-localized expression of a channelrhodopsin and halorhodopsin as one fusion protein and further demonstrated that this new protein can excite or inactivate neurons upon illumination with different wavelengths of light.

Previously, we reported^24^ a system that adopted a highly expressible bacteriorhodopsin (HEBR) system in *E. coli* that served as an expression tag preceding target membrane proteins to enhance target protein functional expression in *E. coli* and achieved a yield of more than 2 mg/mL. In this study, we successfully extended one transmembrane segment from HEBR to fuse with various target M-Rho proteins, including *Halobacterium salinarum* bacteriorhodopsin (HsBR), *Natronomonas pharaonis* halorhodopsin (NpHR), *Haloarcula marismortui* sensory rhodopsin II (HmSRII), and *Haloarcula marismortui* sensory rhodopsin M (HmSRM). In each construct, the optical property, function and stability of the target M-Rho protein were examined and found to be intact or unaltered.

## Results

### Construction of HEBR-assisted M-Rho plasmids for *E. coli* expression

The D94N mutant of *H. marismortui* bacteriorhodopsin I, designated HEBR for short, was constructed as described previously^25^. The yield of HEBR alone reached 60–70 mg/L in culture without any reconstitution or refolding^24^, and the maximum absorbance (Abs-max) of HEBR^26^ was 552 nm, almost identical to the wild type protein, HmBRI. Such an overexpression yield in *E. coli* system inspired the adoption of HEBR as a co-expression tag for other kinds or types of M-Rho proteins to create fusion proteins featuring multi-capability yet with ample expression yield for further creative applications.

**Figure 1:**
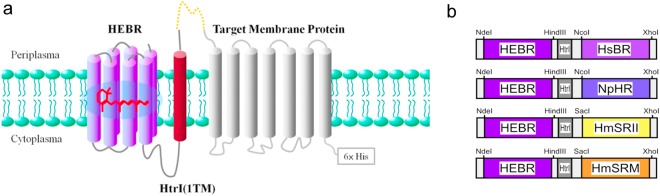
Construction strategy of the HEBR fusion with target protein(s). (**a**) The transmembrane region 1 (TM1) of HmHtrI was introduced between HEBR and the target membrane protein with the N-terminus residing in the periplasmic side; see the Methods for more details. (**b**) Diagrams summarize the tandem expression cassette variants and the designed restriction enzyme sites for the construction of the target microbial rhodopsin.

To fuse and co-express another M-Rho protein with HEBR (Fig. 1a), the C-terminus of HEBR was first extended using the first transmembrane region (TM1), a total of 37-a.a. in length, from HmHtrI. The TM1 was then connected with a flexible loop comprised of Ala-Ser-Ala-Ser-Asn-Gly-Ala-Ser-Ala followed by a designated target M-Rho containing a six-histidine tag appended to its C-terminus. The expression cassettes and restriction enzyme cutting sites for all the constructs in this study were summarized in Fig. 1b. Among them, a) HEBR-HsBR contains a bacteriorhodopsin from *H. salinarum*, which is known to have an extremely low expression efficiency in *E. coli*; b) HEBR-NpHR has a halorhodopsin from *N. pharaonic* that is a light-driven inward chloride transporter; c) HEBR-HmSRII attaches a sensory rhodopsin from *H. marismortui*, which is known to sense wavelengths approximately 498 nm and mediates a photorepellent response; and d) HEBR-HmSRM has a new type of functionally unknown sensory rhodopsin^25^ from *H. marismortui*, which was reported to be extremely unstable after purification.

### Visual colors of purified target proteins and UV-vis absorbance spectra

**Figure 2:**
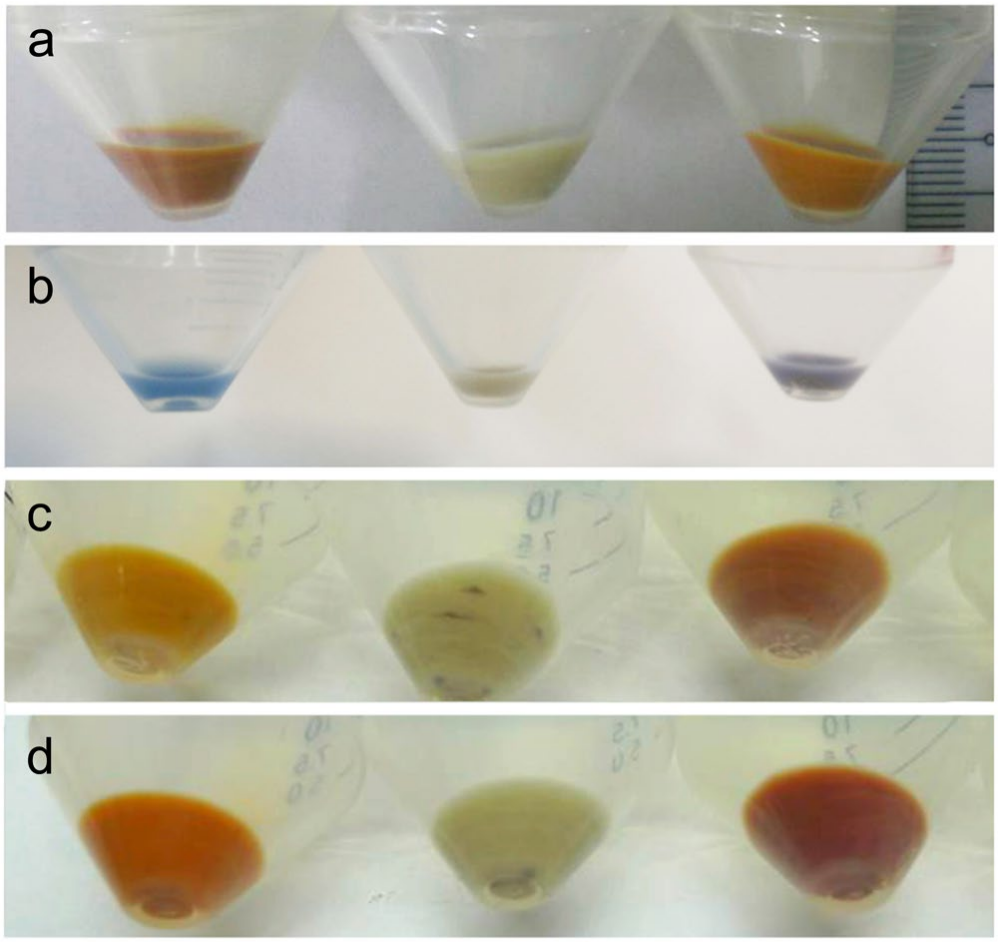
Visual color of *E. coli* cells expressing different fusion proteins. The visual color of the centrifuged *E. coli* cell pellets after induction with IPTG and all-trans retinal. Panel a: HEBR alone (left), HEBR-HsBR without (middle) and with 10 μM all-trans retinal during IPTG-induction (right). Panel b: NpHR alone (left), HEBR-NpHR without (middle) and with all-trans retinal during IPTG-induction (right). The same for the following panels, panel c: HmSRII alone (left), HEBR-HmSRII without (middle), and with retinal (right). Panel d: HmSRM alone (left), HEBR-HmSRM without (middle), and with all-trans retinal (right) during IPTG-induction.

The expression of these four fusion proteins is described in the Methods. Briefly, plasmids of fusion proteins were first transformed into *E. coli* C43, and an overnight single colony was inoculated in LB broth until OD_600_ = 0.4–0.6 before being induced with IPTG to a final concentration of 1 mM and 5–10 μM of all-trans retinal (Sigma, R-2500, USA). After another 4–6 hours of culture, the cells were harvested using centrifugation, and the pellets were observed with unaided eyes for their visual colors (Fig. 2) after removal of the supernatant. The cell pellets expressing different fusion proteins were visually distinguishable by the color difference compared with the target protein alone. Such visual color differences can serve as early indications for successful fusion protein expression. On the practical side, our system paves the way for the potential to develop a screening system to evaluate the expression efficiency of target proteins achieved by the visual color of ITPG-induced *E. coli* cell pellets and can also be applied to search particular color tuning mutants.

All the proteins expressed were first purified as described in the Methods. A final minimum yield of 2 mg/L culture was observed in HEBR-HsBR, while all the other target proteins had yields of more than 4 mg/L culture. Different *E. coli* strains were used (Supplementary Fig. 1) to prove that this fusion strategy was not due to effects of the C43 strain.

**Figure 3:**
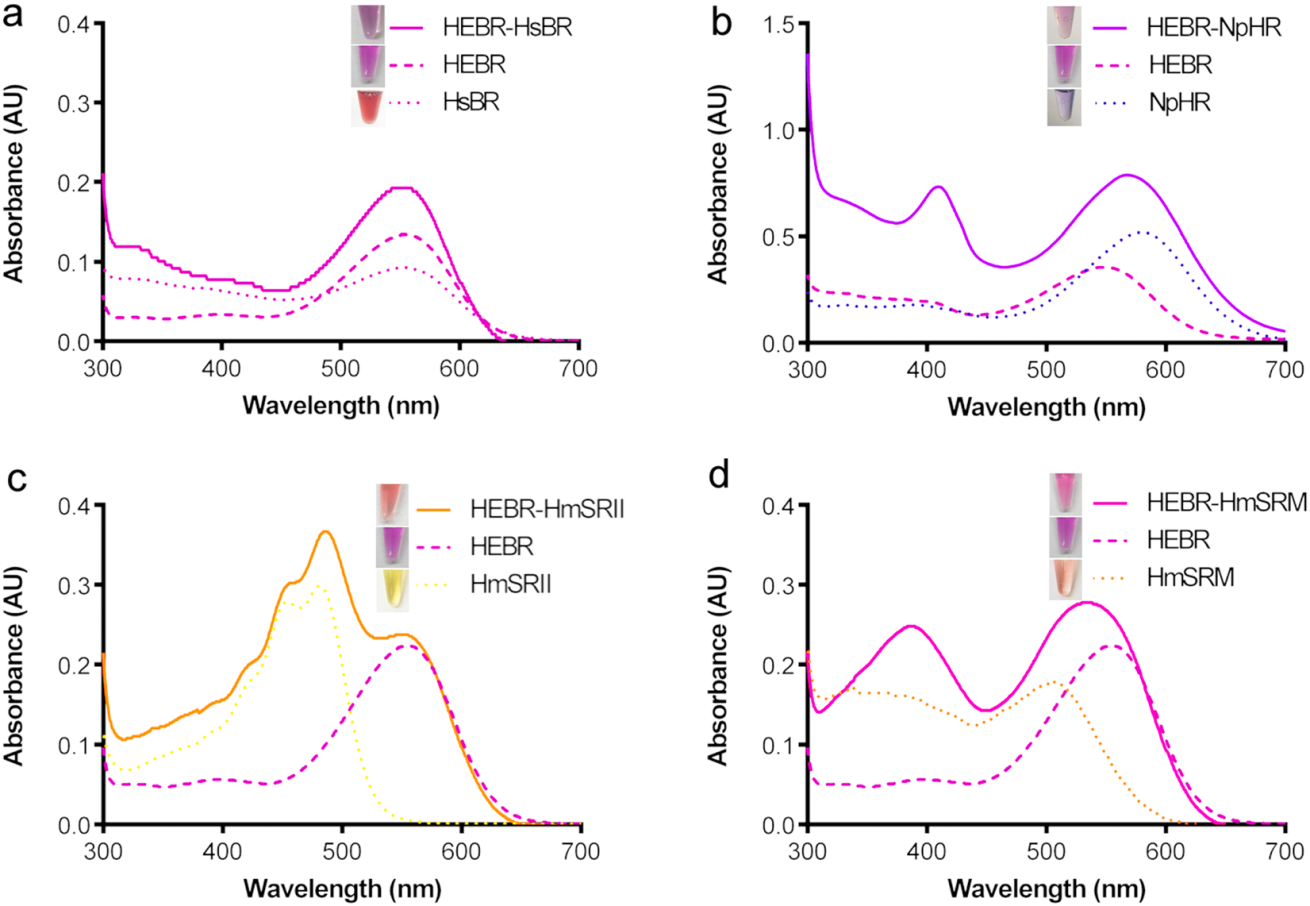
UV-Vis spectrum analysis and visual colors of HEBR fusion proteins. Four separate measurements of absorbance spectra were plotted in the same chart: solely HEBR (bold dashed), target protein (dashed) and fusion protein (solid): (**a**) HEBR-HsBR, (**b**) HEBR-NpHR, (**c**) HEBR-HmSRII and (**d**) HEBR-HmSRM. The visual color of purified proteins (inlet of their corresponding figure). All the samples were prepared in 50 mM MES, 4 M NaCl, pH 5.8, containing 0.05% DDM.

The two primary optical properties are the maximum absorbance (Abs-max) and the kinetics of the photocycle. To test whether the HEBR fusion tag disturbs the Abs-max of the individual target M-Rho in fusion proteins, the purified (Fig. 3, inlets) HEBR-HsBR, HEBR-NpHR, HEBR-HmSRII, and HEBR-HmSRM were subjected to UV-Vis spectrophotometric scanning (Fig. 3a–d). When compared with the Abs-max of singly expressed target M-Rho proteins, the UV-vis scanning profiles of the fusion proteins each showed a pure combination of the scanning profile of a singly expressed M-Rho merged with that of HEBR, thus indicating that the designed fusion tag, HEBR, TM1 of HmHtrI and the fusion loop did not disturb the optical property of either the individual target M-Rho or HEBR itself, in each case.

### Photocycle and light-driven proton assays showed the un-altered function of the target M-Rho

**Figure 4:**
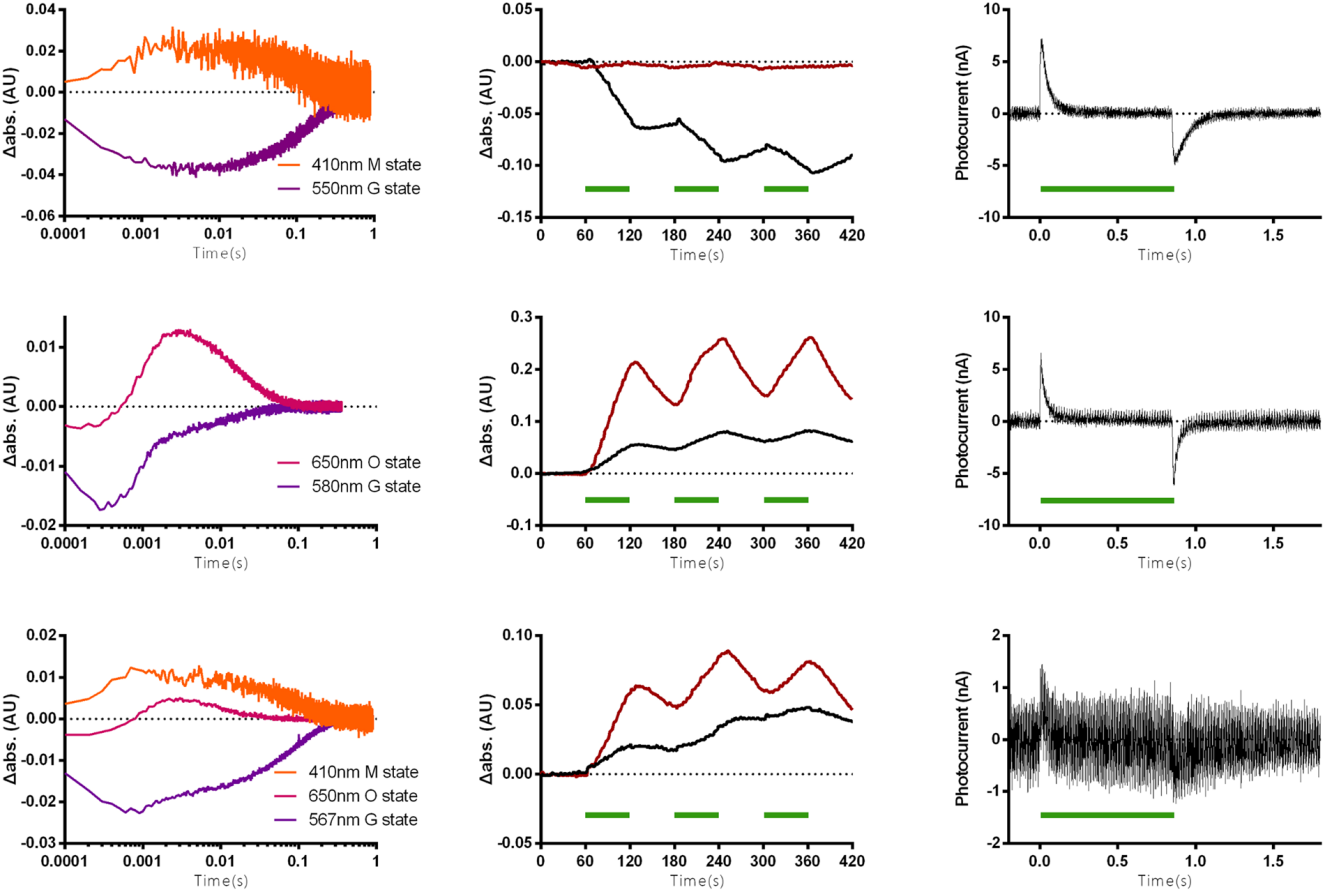
Flash laser-induced photolysis and photocurrent for ion-type HEBR fusion proteins. (**a**) Flash laser-induced absorbance changes in HEBR (top), NpHR (middle), and HEBR-NpHR (bottom) under different wavelengths were recorded. (**b**) Light-driven pH change measurements in *E. coli* expressing HEBR (top), NpHR (middle), and HEBR-NpHR (bottom) without (black) and with (red) the addition of the ionophore CCCP (carbonyl cyanide m-chlorophenyl hydrazone). (**c**) Purified protein-based light-driven photocurrent measurements of HEBR (top), NpHR (middle), and HEBR-NpHR (bottom). Green bars indicate the duration of illumination with 532 nm green laser in all occasions.

The second optical property, the kinetics of the photocycle and the light-induced photocurrent were examined for the HEBR-NpHR fusion protein. In the laser-induced photolysis measurements, the ground state photocycle profile of the HEBR-NpHR fusion protein (Fig. 4a, bottom, 567 nm) showed a pattern resembling that of a merged HEBR (Fig. 4a, top, 550 nm) and NpHR alone (Fig. 4a, middle, 580 nm). The 410 nm M-intermediate that only appeared in HEBR alone and the 650 nm O-intermediate that was only detectable in NpHR were both recorded in the HEBR-NpHR fusion protein, indicating that both HEBR and NpHR were still active.

The functionality of HEBR-NpHR was probed with the whole cell *E. coli* pH and protein-based photocurrent assays that we previously adopted and developed^27^. After expressing HEBR-NpHR in *E. coli*, the cells were centrifuged and resuspended in a non-buffer solution. Upon illumination of cells with a 532-nm laser (Fig. 4b, top-bottom, indicated with green bar), the pH changes in the solution were monitored. In *E. coli* cells expressed with HEBR alone (Fig. 4b top), the illumination induced a pH decrease in each repeat (black line), while those pH changes were eliminated in the presence of an ionophore CCCP (red line). In the cells expressing solely NpHR (Fig. 4b middle), the whole-cell measurement showed the pattern established to indicate light-driven inward chloride pumping action, which is known to create a passive proton influx that consequently leads to an increase in the environmental pH. In the cells expressing the HEBR-NpHR fusion protein (Fig. 4b bottom), it showed a pattern resembling that of NpHR cells alone but is surmised to be a combined pattern of NpHR and HEBR with the NpHR component being dominant. The rationale is that NpHR executes light-driven inward chloride pumping 10-times faster than the light-driven proton outward pumping of HEBR.

In addition to the whole-cell measurements, we also conducted purified protein-based *in vitro* proton pumping measurements using pH sensitive ITO glass to detect the proton flow. HEBR (Fig. 4c, top) showed an outward proton flow, since it is classified as a proton pump. The same phenomenon was observed in NpHR (Fig. 4c, middle), which was reported to undergo a proton circulation on the cytoplasmic side^27^. The same measurement was conducted on the HEBR-NpHR fusion protein (Fig. 4c, bottom) to examine its biological functionalities. In the fused form, a light-triggered proton outward flux signal was observed, indicating that both HEBR and NpHR were functionally intact.

**Figure 5:**
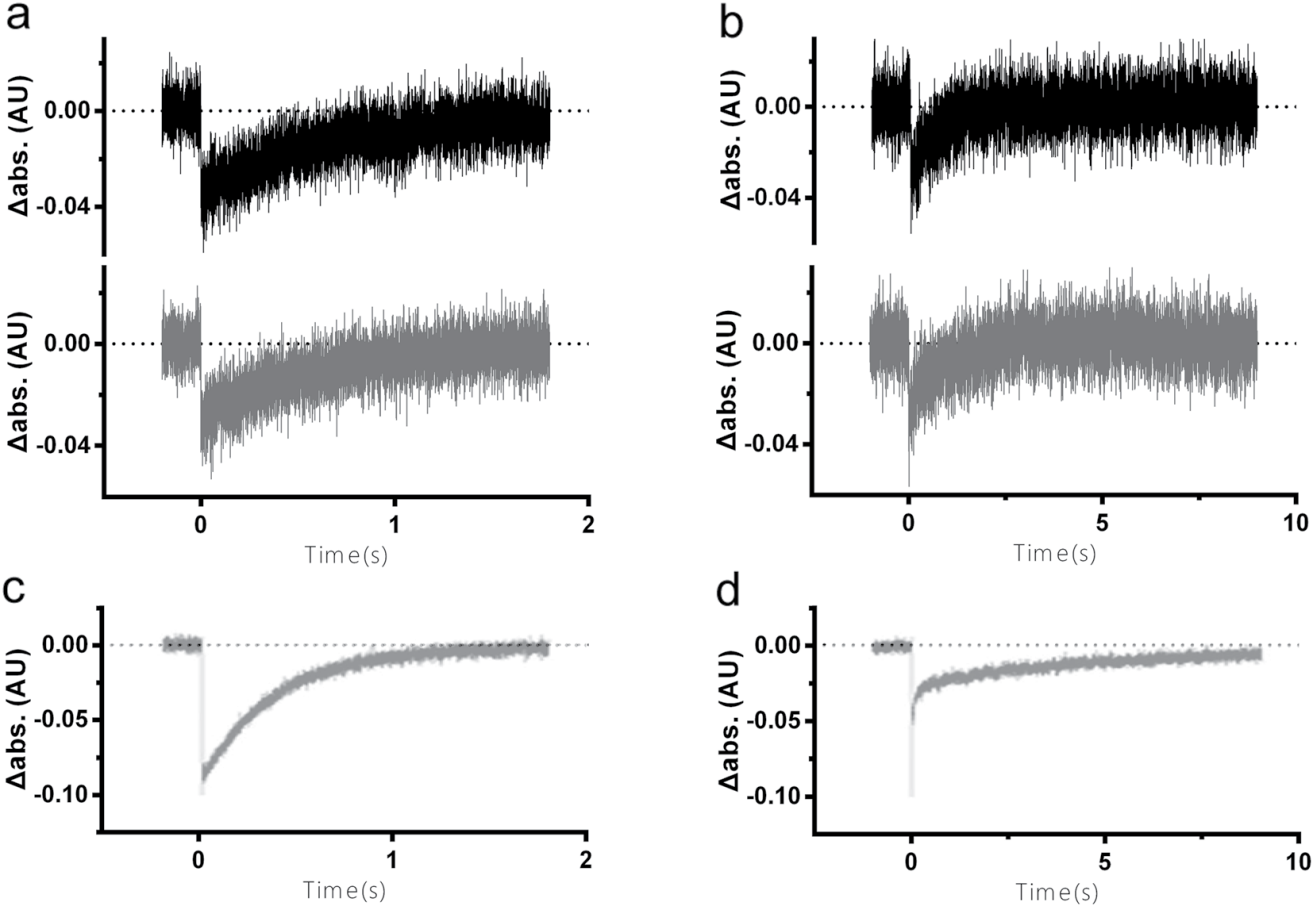
Flash laser-induced photolysis for sensory-type HEBR fusion proteins. Flash laser-induced absorbance change of (**a**) HEBR-HmSRII under the maximum absorbance (Abs-max) of HEBR (550 nm, black line) and that of HmSRII (486 nm, gray line); (**b**) HEBR-HmSRM under the Abs-max of HEBR (550 nm, black line) and that of HmSRM (505 nm, gray line); (**c**) HmSRII under its Abs-max, 486 nm; (**d**) HmSRM under its Abs-max, 505 nm.

Due to the lack of a valid *in vitro* functional assay for sensory rhodopsins, the status of HmSRII and HmSRM was examined with their optical properties. In a flash laser-induced photolysis experiment, the decay and regeneration of the ground state upon light-activation is a solid implication of the stability, integrity, and functionality of sensory rhodopsins^28^. In the case of the fusion rhodopsins, HEBR-HmSRII was observed to complete its photocycle at the time-scale of ~2 seconds when monitored with the Abs max of HEBR (Fig. 5a, upper) and that of HmSRII (Fig. 5a, lower), both patterns being comparable to both HmSRII alone (Fig. 5c) and HEBR alone^26^.

A faster recovery kinetic of 4 seconds in the HEBR-HmSRM protein was observed at both 505 nm and 550 nm (Fig. 5b), which are the Abs-max for HmSRM and HEBR, respectively, in comparison to the ~10 seconds measured in HmSRM alone (Fig. 5d). These results indicated that the target M-Rho retained its original photocycle properties in the fused form. The otherwise unstable HmSRM even showed an accelerated photocycle, from 10 s to 4 s in HEBR-HmSRM form (Fig. 5b,d), in addition to only marginally shifted spectra under extreme conditions of temperatures, salt concentrations, and pH conditions (Supplementary Fig. 2), indicating that the HmSRM was stabilized under such a fusion system. We therefore conclude that sensory type M-Rho can be fused with HEBR to obtain functionally intact proteins.

## Discussion

Microbial rhodopsins have been widely adopted for biotechnological and biophysical applications during the previous decades. They are primarily utilized in optical appliances, therapeutic/medical applications and certain computational purposes^21^, including the most recent optogenetic studies^29^. In this study, we successfully developed an expression strategy using the HEBR system that we previously reported to create a single fusion protein with a combination of two M-Rho proteins. All the fusion proteins were functional and showed reasonable yields.

Our study demonstrated that when two M-Rho proteins were fused together in our HEBR system, both proteins can still conserve their individual functionality and biophysical properties. As described, a study^23^ fused the NpHR from *Natronomonas pharaonis* with a light-gated inward cation channel ChR2 from *Chlamydomonas reinhardtii*, and when expressed in nerve cells, this fusion protein could deactivate and activate nerve fibers upon illumination of ~580 nm or ~480 nm of light, respectively. It should be noted that even though this study showed a Western blot to confirm the expression of the proteins in cells, it lacked further confirmation of direct protein purification, Abs-max determination, photocycle measurements, and direct *in vitro* functionality examinations. However, it clearly showed wavelength-dependent excitation or inhibition effect on nerve activity when expressed in neuron cells, and their results thus supported our findings of the unaltered Abs-max for the individual M-Rho proteins in the fused form.

### The HEBR fusion system in this report provides several advantages

#### First, it offers ample expression efficiency for fused M-Rho target proteins and consequently it enables *in vitro* or application studies

The yield of the whole fusion proteins reached at least 2 mg/L culture in four different types of fusion proteins, including an improvement of HsBR from almost non-expressible in *E. coli* and two sensory rhodopsins. Therefore, HEBR brought fused protein expression to a minimum reasonable expression level regardless of the original expression efficiency of the target M-Rho proteins.

#### Second, M-Rho proteins fused with HEBR retain their biophysical properties

Our data concludes that HEBR shows no alteration in both the photocycle and Abs-max in both ion-type and sensory-type target M-Rho proteins. In the four HEBR fusion proteins studied, all individual target M-Rho conserved their Abs-max and photocycle. Therefore, it can be claimed that HEBR shows no negative effects toward the target fused proteins, at least in this study.

#### Third, the M-Rho proteins fused to HEBR are all still functionally intact

In addition, the light-driven ion transportation functions of BR and HR are conserved in the fused form. At the same time, HEBR does not disturb the otherwise unstable lone sensory type M-Rho. Interactions with the transmembrane region of their corresponding cognate transducers was known to stabilize sensory M-Rho proteins as reported in HsSRI^30,31^, NpSRII^32^ and HmSRM^25^. These results suggest that the HEBR fusion M-Rho might increase or at least maintain the stability of the target proteins.

In summary, based on the success of our previous co-expression and fusion protein study, we combined the previous conclusions and applied them to our HEBR system. We show a universal strategy to overexpress two M-Rho proteins as a single fusion protein, which reaches ample expression efficiency for *in vitro* purification and investigation, while at the same time conserving the individual biochemical and biophysical properties of the two proteins. Therefore, these advantages increase the confidence to design multi-functional M-Rho using our HEBR system for further creative applications.

## Methods

### Bacterial strains and plasmid construction

*Escherichia coli* DH5α was used for cloning. *E. coli* C43(DE3) and *E. coli* C41(DE3) were used to express protein. The rhodopsin and transducer genes were amplified using PCR from the genomic DNA of H. marismortui as described previously^24^. Primers for the first transmembrane domain of HmHtrI (forward, 5′-ATATAAGCTTGCGTCGGCGTCGAACGGCGCGTCGGCGATGACTATTTCTAGTGTC-3′, reverse, 5′-ATATCTCGAGTGCGGCCGCCATGGTGAGCTCGCGTCCCTCAATCGATGCAACATCTTGTGT-3′) were designed by introducing a HindIII restriction enzyme cutting site into the N-terminal and a *Nco*I, *Sac*I, and *Xho*I restriction enzyme cutting site into its C-terminal. The DNA fragments were digested using *Hind*III-*Xho*I and ligated into the pET-21b(+) containing HEBR. The genes of HsBR and NpHR were previously constructed and digested using *Nco*I and *Xho*I. Primers for sensory rhodopsins, HmSRII (forward, 5′-ATATGAGCTCATGGCAACGATAACAACC-3′, reverse, 5′-ATATCTCGAGGTCCCCTGCAACCGCTGT-3′) and HmSRM (forward, 5′-ATATGAGCTCATGGCACAAGAGATCGTT-3′, reverse, 5′-ATATCTCGAGCTTGGCGGGAGCTACGGA-3′) were designed by introducing a *Sac*I restriction enzyme cutting site into the start codon and a *Xho*I restriction enzyme cutting site in opposition to the stop codon. The *Sac*I-*Xho*I DNA fragments were digested and ligated into HEBR-HmHtrI contained in pET-21b(+). Consequently, the vector encoding six histidines at the C terminus, which resulted in the following N- and C-terminal peptide sequences: M—LEHHHHHH, in which the hyphens represent the protein sequence for the gene of interest, and the underlined amino acids represent the introduced *Xho*I site. The accuracy of all the constructs was confirmed using nucleotide sequencing.

### Protein expression and purification

A single colony of transformed *E. coli* C43 (DE3) or *E. coli* C41(DE3) cells was inoculated in LB (Luria–Bertani) medium supplemented with 50 μg/mL of ampicillin and incubated at 37 °C overnight. For large-scale protein expression, a 1:100 (v/v) dilution of the overnight culture was added to fresh LB/ampicillin media and incubated at 37 °C. When the OD_600_ of the culture reached 0.4–0.6, IPTG (isopropyl β -D-1-thiogalactopyranoside; final concentration 1 mM) and all-trans retinal (final concentration 5–50 μM) were added for induction. Following subsequent incubation for 4–6 h in the dark, the cells were collected using centrifugation at 6750 × g for 10 min at 4 °C (Hitachi CR-21, R10A3). The cells collected were resuspended in buffer A (50 mM Tris/HCl, 4 M NaCl, 14.7 mM 2-mercaptoethanol and 0.2 mM PMSF, pH 7.8) and broken by ultrasonic processing (S-3000; MISONIC). To separate the membrane fraction, total cell-extract centrifugation was performed at 6750 × g for 10 min at 4 °C (Hitachi CR-21, R20A2). The supernatant was centrifuged at 169,538 × g for 1 h at 4 °C (Hitachi CP80WX, P70AT). The sediment was dissolved in buffer B (buffer A supplemented with 1% DDM (n-dodecyl-β -D-maltoside) for at least 12 h at 4 °C, followed by centrifugation at 32816 × g for 45 min at 4 °C (Hitachi CR-21, R20A2) to separate the detergent-soluble fraction. Solubilized proteins were purified by affinity purification using the Ni-NTA (Ni2+-nitrilotriacetate) method. The detergent-soluble solution containing 20 mM imidazole was incubated with Ni-NTA agarose at 4 °C for 6–8 h on an orbital shaker. It was transferred to a chromatography column and washed with buffer C (buffer A with 0.05% DDM and 50 mM imidazole). The target proteins were eluted with buffer D (buffer A with 0.05% DDM and 250 mM imidazole). The purified proteins were concentrated and exchanged into buffer E (50 mM MES, 4 M NaCl and 0.05% DDM, pH 5.8) with a protein concentrator (Millipore, Amicon, cut-off size of 30 kDa).

### UV/Vis spectrum analysis and stability analysis

The absorption spectroscopy and stability analysis of proteins were measured using UV/Vis spectroscopy as described previously^26^. Briefly, the purified and concentrated proteins were diluted 100-fold into different pH value (KCl, citric acid, phosphate, Tris or NaHCO3, each at 100 mM concentration), salt concentration (7.81 mM to 4 M NaCl) and temperatures (4 °C to 64 °C) buffers under red dim light before absorption spectra measurements were determined (U1900 UV/Vis, Hitachi, Japan). The results were used to evaluate the stability of the proteins in different environments. All the measurements were performed at 25 °C.

### Flash-laser-induced photocycle measurements

Flash-induced absorption transients were monitored using a flash-photolysis system designed by our group and described previously^25^. The flash laser was a Nd-YAG laser (532 nm, 6 ns pulse, 40 mJ). The purified proteins were suspended in buffer E (50 mM MES, 4 M NaCl and 0.05% DDM, pH 5.8) to reach 0.3 at OD_λmax_, and the transient absorbance changes were recorded at selected wavelengths. The curves represent the loss and recovery of the absorbance at the indicated wavelength upon green laser (532 nm) excitation. Transients were collected and averaged for each measurement. All the measurements were performed at 25 °C.

### Light-driven photocurrent measurements

A slight modification of the electrochemical cell designed by Chu *et al*.^33^ was used for light-driven photocurrent measurements, and a detailed description can be found in our previous study^27^. Briefly, a 0.5-W 532-nm continuous laser was used to stimulate M-Rho located in the photochemical cell, which was composed in the following order: ITO-coated glass slide, sample chamber, dialysis membrane, blank solution chamber, and then another ITO-coated slide. The two ITO-coated slides were connected by a wire and a signal amplifier (SR570, Stanford Research Systems, Sunnyvale, CA) for photocurrent measurements. For each measurement, 64 trials were averaged. The purified proteins were dialyzed against a non-buffer solution (10 mM NaCl and 0.02% DDM).

## Electronic supplementary material

Supplementary data
