# Supplementary material for: Overexpression of Different Types of Microbial Rhodopsins with a Highly Expressible Bacteriorhodopsin from Haloarcula marismortui as a Single Protein in E. coli
Source: Sci Rep. 2018 Sep 19;8:14026. doi: 10.1038/s41598-018-32399-x (PMC6145879; doi:10.1038/s41598-018-32399-x)
Supplement: Supplementary file 1 — Supplementary data [file 41598_2018_32399_MOESM1_ESM.docx]

**Supplementary Materials**

**Title**
Overexpression of Different Types of Microbial Rhodopsins with a Highly Expressible Bacteriorhodopsin from *Haloarcula marismortui* as a Single Protein in *E. coli*

**Authors**Cheng-Hong Tu^1,+^, Hsiu-Ping Yi^1,+^, Shiang-Yuang Hsieh^1^, Hong-Syuan Lin^1^, and Chii-Shen Yang^1,*^

^1^Department of Biochemical Science and Technology, National Taiwan University, Taipei, 10616, Taiwan
^*^Chii-Shen Yang: [chiishen@ntu.edu.tw](mailto:chiishen@ntu.edu.tw)
**^+^**These authors contributed equally to this work.


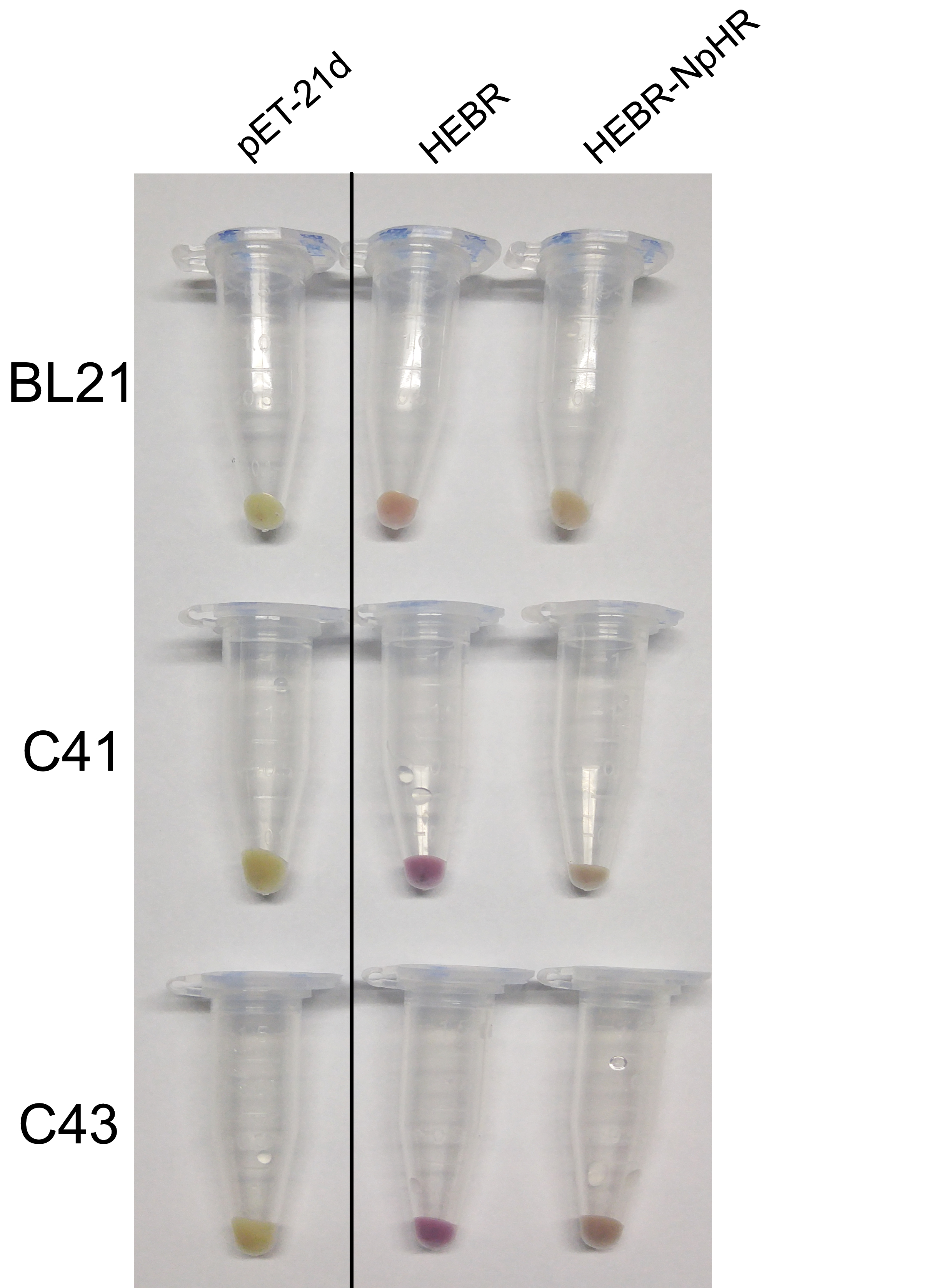


**Supplementary Figure 1. Expression of HEBR-NpHR in three different strains of *E. coli***

The visual colors of centrifuged *E. coli* cell pellets after being transformed and expressed pET-21d vector alone (left), HEBR (middle), and HEBR-NpHR. Three different *E. coli* strains adopted in this study were indicated in each row. Top row: *E.coli* strain BL21; middle row: *E.coli* strain C41; bottom row: *E.coli* strain C43. After being transformed with indicated plasmids, all cells were grew to OD_600_ of 0.5 before induced with 10 mM IPTG and *all-trans* retinal. Comparison of different *E.coli* strains expressing empty plasmid pET-21d, HEBR alone , and HEBR-NpHR concluded HEBR-fusion-NpHR was consistently expressed across different strains *E. coli* cells with only minor variation in efficiencies.


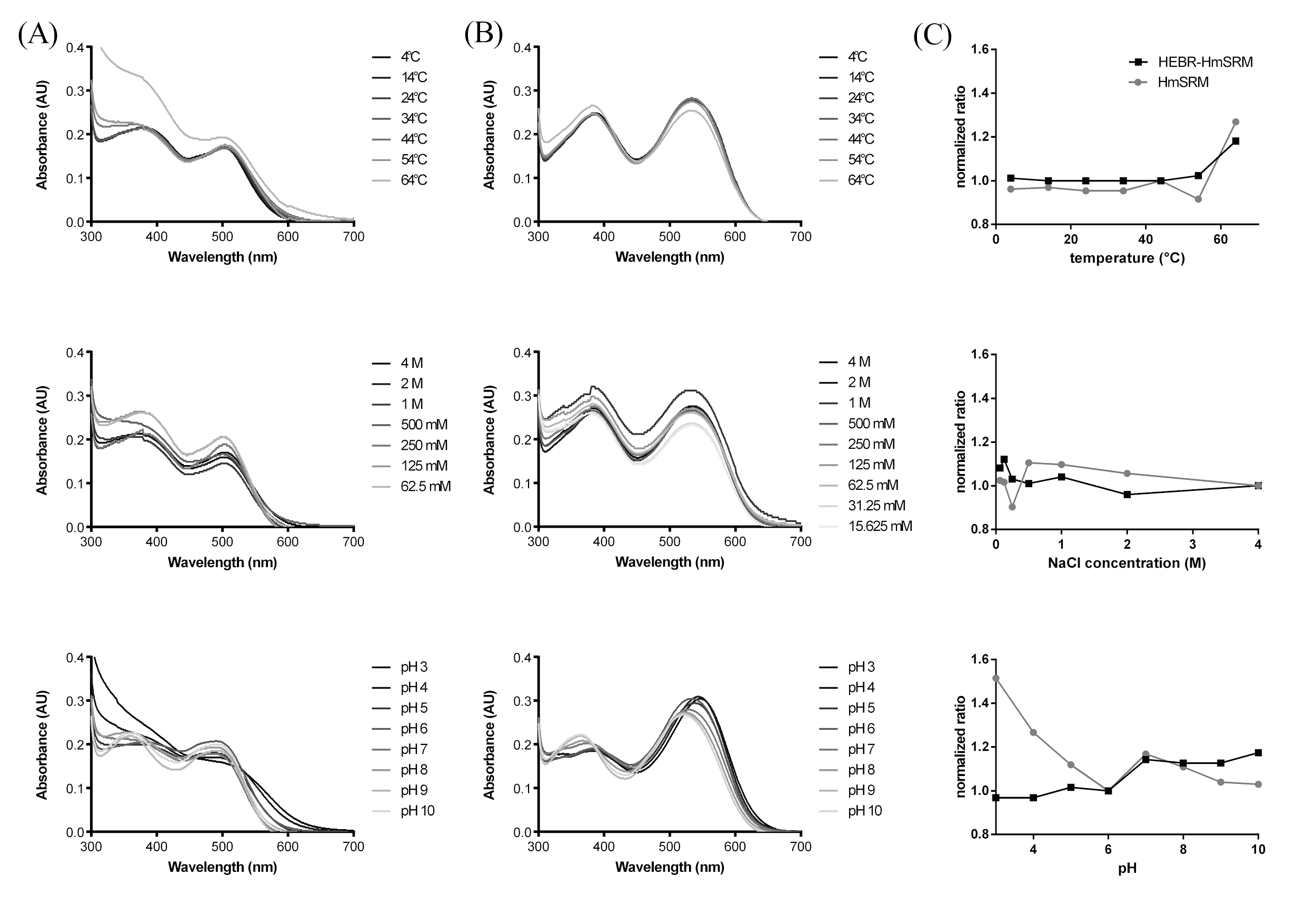


**Supplementary Figure 2. Protein stability of purified HmSRM alone and HEBR-HmSRM.**

Sensory rhodopsin M (HmSRM) from *Haloarcula marismortui* were either alone or being constructed to fuse on the C-terminus of HEBR. After purification, absorbance spectrum of (A) HmSRM alone, and (B) HEBR-HmSRM were measured under three different conditions: temperatures from 4℃ to 64℃ (top), different NaCl concentrations from 62.5mM to 4 M (middle), and pH ranged from 3 to 10 (bottom). (C) The ratios of the M state of HmSRM (385nm) to its ground state (504nm for HmSRM, 534nm for HEBR-HmSRM) were first calculated, and those ratios were further normalized by those values under known optimal environments of *H. marismortui* under 44℃, 4M NaCl, and pH 6. Both HEBR-HmSRM (black square) and HmSRM (grey dot) were shown. Those results concluded the HmSRM showed higher stability when fussed with HEBR.
